# Phosphate and Cardiovascular Disease beyond Chronic Kidney Disease and Vascular Calcification

**DOI:** 10.1155/2018/3162806

**Published:** 2018-04-08

**Authors:** Sinee Disthabanchong

**Affiliations:** Division of Nephrology, Department of Medicine, Faculty of Medicine, Ramathibodi Hospital, Mahidol University, Bangkok, Thailand

## Abstract

Phosphate is essential for life but its accumulation can be detrimental. In end-stage renal disease, widespread vascular calcification occurs as a result of chronic phosphate load. The accumulation of phosphate is likely to occur long before the rise in serum phosphate above the normal range since several observational studies in both general population and early-stage CKD patients have identified the relationship between high-normal serum phosphate and adverse cardiovascular outcomes. Consumption of food high in phosphate increases both fasting and postprandial serum phosphate and habitual intake of high phosphate diet is associated with aging, cardiac hypertrophy, endothelial dysfunction, and subclinical atherosclerosis. The decline in renal function and dietary phosphate load can increase circulating fibroblast growth factor-23 (FGF-23) which may have a direct impact on cardiomyocytes. Increased FGF-23 levels in both CKD and general populations are associated with left ventricular hypertrophy, congestive heart failure, atrial fibrillation, and mortality. Increased extracellular phosphate directly affects endothelial cells causing cell apoptosis and vascular smooth muscle cells (VSMCs) causing transformation to osteogenic phenotype. Excess of calcium and phosphate in the circulation can promote the formation of protein-mineral complex called calciprotein particles (CPPs). In CKD, these CPPs contain less calcification inhibitors, induce inflammation, and promote VSMC calcification.

## 1. Introduction

The discovery of phosphorus occurred by accident in 1669 when a German alchemist named Hennig Brand boiled down 60 buckets of urine in search of the “philosopher's stone,” a compound that would turn ordinary metals into gold. The discovered compound glowed in the dark in pale-green color, self-ignited and blew up into flame. He named the compound “phosphorus,” which was taken from the Greek word meaning “bearer of light” [1]. Due to the high reactivity, phosphorus is never found as free element. White phosphorus has been used in manufacturing bombs and red phosphorus is used to make the strike plate of match boxes. The common use of phosphorus in the form of phosphoric acid nowadays is in the fertilizer industry.

Phosphorus is essential for life and exists in the body as phosphate. Phosphates are components of RNA, DNA, adenosine triphosphate (ATP), cell membrane, and bone. An average adult contains approximately 700 gram of phosphorus which is the result of an intake and excretion of 1-2 grams per day. Phosphate is excreted mostly in the urine. Only 0.1% of body phosphate circulates in the blood.

**Figure 1:**
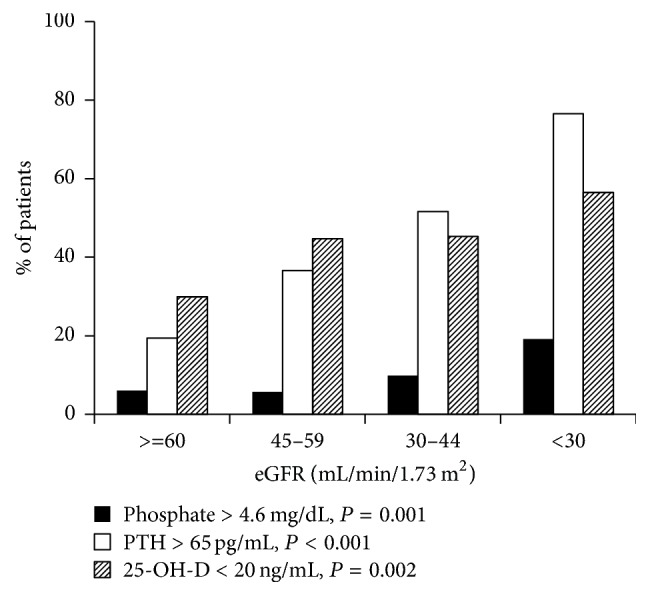
*Prevalence of hyperphosphatemia according to kidney function*. *P* values represent the significance of trend.* Reuse with permission from Chartsrisak et al. [3].*

Despite its importance, the accumulation of phosphate can produce deleterious effects. Such example can be seen in end-stage renal disease patients when widespread vascular and soft tissue calcifications occur as a result of chronic phosphate accumulation. In early stages of chronic kidney disease (CKD), serum phosphate is normally maintained within the normal range owing to the compensatory increase in fibroblast growth factor-23 (FGF-23) and parathyroid hormone up until the estimated glomerular filtration rate (eGFR) reaching 30 mL/min/1.73 m^2^. Beyond this point hyperphosphatemia begins to develop [2, 3] (Figure 1). However, the accumulation of phosphate occurs long before the rise in serum phosphate above the upper normal limit since several observational studies in both general population and early-stage CKD patients have identified the relationship between high-normal serum phosphate and adverse cardiovascular outcomes. The following review will focus on the role of phosphate accumulation in cardiovascular disease (CVD) beyond CKD and vascular calcification.

## 2. Serum Phosphate and Outcomes

**Figure 2:**
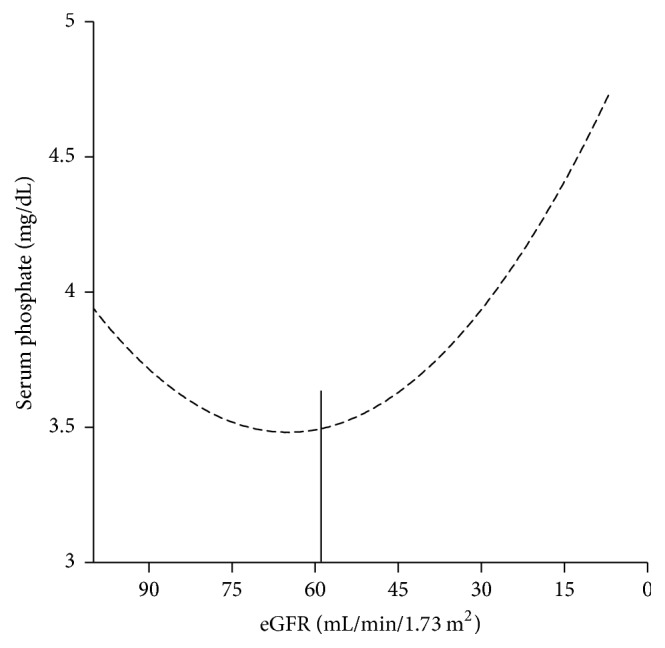
*Serum phosphate according to kidney function*. Vertical line represents the change in slope.* Modified from Chartsrisak et al. [3].*

In CKD, the gradual increase in serum phosphate can be observed since the beginning of stage 3 [2] (Figure 2). Several studies in early-stage CKD patients have identified the relationship between increased serum phosphate but still within the normal range with adverse cardiovascular and renal outcomes and overall survival [5–8]. The reported thresholds of serum phosphate in nondialysis CKD stages 2–5 patients that have been shown to predict adverse outcomes ranged between 3.5 and 4.6 mg/dL (Table 1). These data suggest that phosphate accumulation occurs since early stages of CKD prior to the development of hyperphosphatemia. More interestingly, the relationship between high-normal serum phosphate and adverse outcomes extends beyond CKD population. Among population with preserved renal function (normally defined as eGFR >= 60 mL/min/1.73 m^2^), the increase in serum phosphate not only displays a relationship with makers of atherosclerosis, for example, vascular and valvular calcifications, but also predicts atherosclerotic and nonatherosclerotic cardiovascular events and mortality [9–19]. The reported thresholds of serum phosphate for adverse outcomes were lower than CKD population and ranged between 2.5 and 3.8 mg/dL (Table 1). Since two major factors that determine serum phosphate level are dietary phosphate and urinary excretion, it is likely that high dietary phosphate is one of the mediators of such relationship.

## 3. Dietary Phosphate

**Figure 3:**
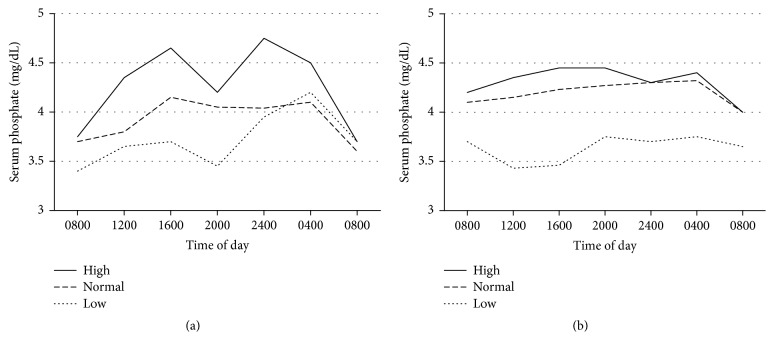
*Serum phosphate concentrations throughout the day in healthy controls (a) and CKD patients (b).* High phosphate = 2500 mg/day; normal phosphate = 1500 mg/day; low phosphate = 1000 mg/day plus lanthanum carbonate. Adapted from Ix et al. [4]. Reuse under the copyright license of free access article from American Society of Nutrition. https://nutrition.org/publications/guidelines-and-policies/license/.

The study that included both healthy and CKD subjects revealed a circadian rhythm of serum phosphate after ingestion of phosphate-rich meal (Figure 3) [4]. Serum phosphate is lowest in the morning and highest at 4 pm and midnight. Consumption of 1500 mg/day (normal phosphate diet) and especially 2500 mg/day of phosphate (high phosphate diet) resulted in a higher fasting and peak serum phosphate compared to consumption of 1000 mg/day of phosphate plus lanthanum carbonate (low phosphate diet). This circadian rhythm also presents in CKD patients but is much less pronounced. Another study in both healthy humans and rats with varying degree of kidney function revealed similar findings. A more rapid elevation of serum phosphate was observed in humans and rats with higher levels of kidney function [20]. These data confirmed that high phosphate diet results in a substantial increase in both fasting and postprandial serum phosphate. Therefore, a habitual intake of high dietary phosphate is likely to chronically elevate serum phosphate, eventually resulting in unfavorable outcomes mentioned above. The study that examined a relationship between increased serum and dietary phosphate with biochemical markers of aging revealed significant associations with telomere length, DNA methylation content, and chronological age [21]. In this study, dietary derived phosphate was closely related to the amount of red meat consumption. Moreover, the relationship between serum phosphate (within the normal range) and dietary phosphate with left ventricular mass was observed in early stages of CKD patients as well as in individuals with preserved renal function [22, 23]. In a large cohort of healthy subjects with no known CVD, dietary phosphate intake >1 gram/day was significantly associated with greater left ventricular mass after adjustment for confounders. Acute dietary phosphate load in healthy adult subjects can impair endothelial-dependent flow-mediated dilatation which may predispose to future atherosclerotic CVD [24, 25]. The associations between increased serum phosphate and increased consumption of dietary phosphate additives with carotid intima-media thickness also exist [26, 27] In addition, dietary phosphate load can also increase FGF-23 concentration and the increase in FGF-23 has been linked to cardiac hypertrophy and adverse cardiovascular outcomes [28–30].

## 4. Fibroblast Growth Factor-23

FGF-23 is produced by osteoblasts and osteocytes in the bone under physiological condition. In the kidney, FGF-23 binds to FGF receptor in the proximal tubule in the presence of coreceptor klotho resulting an inhibition of proximal tubular phosphate reabsorption and a suppression of 1,25-dihydroxy vitamin D synthesis [31]. In CKD, FGF-23 levels increase since stage 2 and continue to rise as CKD progresses. In CKD stages 5-5D, FGF-23 levels are normally several hundred folds above the normal range [2, 32]. In healthy subjects, FGF-23 increases after hours of dietary phosphate load; however, a 4-hour intravenous infusion of phosphate does not alter FGF-23 level at 6 hours, whereas chronic phosphate infusion results in an increase in FGF-23 at 24 hours [28–30, 33, 34]. These data suggest a rather indirect influence of phosphate on FGF-23 secretion. The situation may be somewhat different in CKD when these patients are predisposed to phosphate accumulation due to reduced renal function. To date, the exact relationship between phosphate and FGF-23 in CKD remains unclear. In epidemiological studies, both eGFR and serum phosphate correlate closely with FGF-23 levels [35, 36]. Similar to healthy subjects, dietary phosphate load in subjects with impaired renal function results in an increase in circulating FGF-23 [37]. However, both experimental and epidemiological studies have confirmed the increase in circulating FGF-23 since CKD stage 2 prior to any significant accumulation of phosphate. This early increase in FGF-23 drives a dip in serum phosphate from baseline as a result of heightened urinary phosphate excretion (Figure 2) [2, 38]. These evidences indicate that, initially, the stimuli for FGF-23 secretion is the decline in eGFR followed by the accumulation of phosphate in the later period.

Several studies in populations with preserved renal function and early CKD have linked FGF-23 to left ventricular hypertrophy and decreased left ventricular ejection fraction [39–42]. Increased circulating FGF-23 has also been shown to predict incident and worsening heart failure, atrial fibrillation, cardiovascular events, and mortality [40, 43–55]. One of the important evidences that connects high circulating FGF-23 to abnormal cardiac structure and function is the direct effect of FGF-23 on cardiomyocytes. Pathological level of FGF-23 can induce cardiomyocyte hypertrophy through its binding to FGF receptor-4 in a klotho-independent manner [56, 57]. Further evidence also indicates that the stressed myocardium under pressure or volume overload can also produce FGF-23 resulting in a marked increase in FGF-23 level [58]. These data suggest that the stimuli for FGF-23 secretion include not only diminished renal function and phosphate load but also myocardium under stress. The latter explains the rather consistent relationship between increased FGF-23 levels with cardiac hypertrophy and heart failure in the population with preserved renal function. Furthermore, the antagonistic effect of FGF-23 on 1,25-dihydroxyvitamin D can also trigger renin-angiotensin-aldosterone system resulting in an increase in sodium reabsorption [54, 59]. Recent evidence also suggests that FGF-23 is a negative regulator of erythropoiesis and may promote inflammation [60–62].

## 5. Extracellular Phosphate and Cytotoxicity

Increased extracellular phosphate can induce vascular smooth muscle cell (VSMC) transformation to osteogenic phenotype [63]. These osteogenic VSMCs can release matrix vesicles in a similar fashion to osteoblasts but with less calcification inhibitor, matrix-gla protein. Dying VSMCs also form apoptotic body. Both matrix vesicles and apoptotic bodies have the ability to concentrate and crystalize calcium and phosphate in the preparation for mineralization [64]. In addition to the effect on VSMCs, increased extracellular phosphate can also induce endothelial cell apoptosis [65]. Recent knowledge on extracellular phosphate and cytotoxicity is derived from works related to the formation of protein-mineral complex or calciprotein particles (CPPs). First, fetuin-A, a naturally occurring calcification inhibitor, binds and sequesters calcium and phosphate forming primary CPPs. Primary CPPs then undergo topological rearrangement to form a more stable structure referred to as secondary CPPs. These CPPs exist as colloids and do not precipitate spontaneously [66]. Serum CPP levels increase as kidney function declines and correlate independently with serum phosphate. CPPs can be detected since early stages of CKD when baseline serum phosphate is still within the normal range [67, 68]. At first, CPPs were believed to play a protective role in sequestering and inhibiting calcium-phosphate crystal growth. However, several observational studies have identified the relationship between increased circulating CPPs, especially secondary CPPs, with inflammation, coronary artery calcification, aortic stiffness, and mortality [68, 69]. It is possible that CPPs are bioactive ligand that can induce cellular toxicity. Indeed, in vitro studies have shown that secondary CPPs (not primary CPPs) can induce inflammation and promote osteogenic differentiation of VSMCs [70, 71]. The recent study has also identified the difference between CPPs from healthy subjects and CPPs from CKD patients. Secondary CPPs from CKD patients have lower levels of calcification inhibitors, fetuin-A, and Gla-rich protein, with increased mineral maturation. These secondary CPPs are readily taken up by VSMCs and induce vascular calcification [72].

In conclusion, phosphate accumulation produces detrimental effects on cardiovascular system resulting in poor patient outcomes. The accumulation of phosphate occurs long before the rise in serum phosphate above the normal range. High phosphate diet can increase serum phosphate and FGF-23. FGF-23 has a direct effect on cardiac myocytes causing myocardial hypertrophy. Increased extracellular phosphate is toxic to endothelial cells, promotes the formation of CPPs, and induces VSMC transformation to osteogenic phenotype.

## Figures and Tables

**Table 1:** Thresholds of serum phosphate for cardiovascular events and mortality in early-stage CKD and general populations.

Studies (year)	Populations	Number	Serum phosphate (mg/dL)	Outcomes
Kestenbaum et al. (2005)	Women: Cr ≥ 1.2 mg/dL Men: Cr ≥ 1.5 mg/dL	3490	>=3.5	All-cause mortality
Bellasi et al. (2011)	CKD stages 3–5	1716	>=4.3	Combined ESRD and all-cause mortality
Chartsrisak et al. (2013)	CKD stages 2–4	466	>4.2	Combined ESRD and all-cause mortality
McGovern et al. (2013)	CKD stages 3–5	13292	>=4.6	Combined CV events and all-cause mortality
McGovern et al. (2013)	CKD stages 1-2	20356	>=3.86	Combined CV events and all-cause mortality
McGovern et al. (2013)	eGFR >= 90, no proteinuria	24184	>=3.86	Combined CV events and all-cause mortality
Tonelli et al. (2005)	Previous acute MI, eGFR >= 60 mL/min	4127	>=2.5	All-cause mortality
			>=2.5	Combined fatal and non-fatal CV events
Dhingra et al. (2007)	eGFR >= 60 mL/min	3676	>=3.2	Incident CVD
Foley et al. (2008)	97% has eGFR >= 60 mL/min	13822	>=3.8	All-cause mortality
Larsson et al. (2010)	Men, eGFR >= 60 mL/min	2176	>=2.8	All-cause mortality
Chang and Grams (2014)	95% has eGFR >= 60 mL/min	12984	>3.5	All-cause mortality
			>3.5	CV mortality

CKD = chronic kidney disease; ESRD = end-stage renal disease; CV = cardiovascular; CVD = cardiovascular disease; eGFR = estimated glomerular filtration rate; MI = myocardial infarction.
